# The social and physical workplace environment and commute mode: A natural experimental study

**DOI:** 10.1016/j.pmedr.2020.101260

**Published:** 2020-11-28

**Authors:** Richard Patterson, David Ogilvie, Jenna Panter

**Affiliations:** MRC Epidemiology Unit and Centre for Diet and Activity Research (CEDAR), University of Cambridge, Cambridge CB2 0QQ, UK

**Keywords:** Active travel, Active commuting, Physical activity, Workplace facilities, Workplace, Environment, Natural experiment, Walking, Cycling

## Abstract

Despite strong evidence for health benefits from active travel, levels remain low in many countries. Changes to the physical and social workplace environment might encourage active travel but evaluation has been limited.

We explored associations between changes in the physical and social workplace environment and changes in commute mode over one year among 419 participants in the Commuting and Health in Cambridge study.

In adjusted analyses, an increase in the presence of one physical characteristic (e.g. bicycle parking or shower facilities) was associated with a 3.3% (95% confidence interval 1.0–5.6) reduction in the proportion of commutes by private motor vehicle and a 4.4% (95% CI 1.2–7.7) increase in the proportion of trips including active modes among men. These associations were not seen in women.

A change to a more favourable social environment for walking or cycling among workplace management was associated with an increased proportion of commutes including active modes in women (4.5%, 95% CI 1.4–7.5) but not men. However, in both genders a change to more a favourable social environment for cycling among colleagues was associated with a reduced proportion of commutes by exclusively active modes (−2.8%, 95% CI −5.0 to −0.6).

This study provides longitudinal evidence for gender differences in the associations between workplace environment and commute mode. A more supportive physical environment was associated with more active commuting in men, while the social environment appeared to have more complex associations that were stronger among women.

## 1 Introduction

Physically active travel modes are associated with health benefits when compared with car use (Dinu et al., 2019, Panter et al., 2018, Patterson et al., 2020). This is the case for walking, cycling and combinations of walking or cycling with other modes, such as public transport (Celis-Morales et al., 2017, Dinu et al., 2019, Panter et al., 2018, Patterson et al., 2019). Despite these benefits, active travel remains stubbornly low in many countries, especially for commuting. For example, in England and Wales approximately 15% of commuters in the 1991, 2001 and 2011 Censuses walked or cycled (Goodman, 2013). However, this overall statistic masks considerable variation. For example, Cambridge saw active commuting increase from 44% to 49% of commuters between 2001 and 2011, largely accounted for by cycling (Goodman, 2013). Although shopping and education trips are more likely to be walked or cycled than commutes, levels of active travel for these trips have also plateaued (Department for Transport, 2018).

Changes to the social and physical environment have been promoted as a way to encourage physical activity; these include changes to the workplace environment, which can influence behaviour such as commuting (National Institute for Health and Care Excellence, 2018, National Institute for Health and Care Excellence, 2008). Working in an environment favourable for walking has been associated with greater physical activity (Marquet et al., 2020). Systematic reviews of workplace interventions to increase active commuting have predominantly found these to be effective, but were limited by the quality of available evidence (Hosking et al., 2010, Petrunoff et al., 2016a, Scheepers et al., 2014). Much existing research lacked a control group and/or was of cross-sectional or ecological design, limiting attempts to draw causal inference (Petrunoff et al., 2016a, Scheepers et al., 2014). Specific characteristics associated with commuting behaviour include: showering or changing facilities (Heinen et al., 2013), cycle storage (Heinen et al., 2013, Hipp et al., 2017), financial incentives (Martin et al., 2012, Thøgersen, 2009), restricted car parking (Knott et al., 2019, Petrunoff et al., 2015), having a workplace travel plan (Petrunoff et al., 2015), incentives to walk, cycle or use public transport (Hipp et al., 2017), provision of workplace walking maps (Hipp et al., 2017) and having co-workers who actively commute (Kaczynski et al., 2010). Some studies created a summary score of the number of characteristics to encourage walking or cycling that were available and found higher active commuting in those with more favourable workplaces (de Geus et al., 2008, Porter et al., 2019). However, several other studies have failed to find associations between physical and/or social workplace characteristics and active commuting (Bopp et al., 2012, de Bourdeaudhuij et al., 2005, de Geus et al., 2008, Handy and Xing, 2011).

This study used a natural experimental approach to explore associations between changes in the physical and social workplace environment and changes in the proportion of commute trips by motorised and active modes, after accounting for individual characteristics and attitudes to personal travel.

## 2 Methods

### 2.1 Sample

Data were from the Commuting and Health in Cambridge study, a longitudinal study of adults aged 16 years and over who worked in Cambridge, UK. Participants completed a postal questionnaire about commuting practices, individual characteristics and workplace characteristics in 2011 and one year later in 2012. Further details of participant recruitment and data collection have been published previously (Ogilvie et al., 2016). From 756 participants in full-time employment in 2011 (baseline), 529 (70%) provided data and were also in full-time employment in 2012 (follow-up). Restricting the analysis to those with complete data for all covariates at both time-points resulted in a final sample of 419 individuals (55% of all baseline participants).

### 2.2 Workplace environment

Participants were asked about the presence of nine predominantly physical characteristics of their workplace, e.g. bicycle racks (full list in Supplemental Table S1). For all physical characteristics except for car parking the available responses were ‘yes’, ‘no’ and ‘don’t know’; for these analyses, we assumed that participants who were unaware of a facility were unlikely to be using it and collapsed ‘no’ and ‘don’t know’ to produce a binary variable. Responses to the question about workplace car parking were dichotomised into those with access to workplace parking (free or paid-for) and those without. The sum of the available physical workplace characteristics was calculated, combining the absence of workplace parking with the presence of the other items, such that a higher score indicated a workplace potentially more favourable for active commuting and less so for car commuting. The change in this summary score between baseline and follow-up was calculated for each participant.

The workplace social environment with respect to walking, cycling and driving was assessed by the level of agreement with five statements about how colleagues and management travelled to work (Supplemental Table S1). Change was classified such that zero indicated no change in attitude; a positive score indicated increased agreement (or reduced disagreement) with the statement, i.e. a more favourable social environment for a given travel mode; and a negative score indicated increased disagreement (or reduced agreement) and therefore less a favourable social environment. Scores potentially ranged from −4 to +4, with the extreme values indicating a change from strong agreement to strong disagreement and vice versa. Change in each of these variables was considered continuous with associations assumed to be linear.

### 2.3 Commute mode

Commute mode for the last seven days was recorded separately for journeys to work and those from work. Available options were: guided bus; other bus or coach; train or underground; car, taxi or van; motorcycle or moped; bicycle; walking; and other. The proportions of all commute journeys that were made exclusively by private motor vehicle (car, taxi, van, motorcycle or moped), that were made exclusively by active modes (walking and/or bicycle), and that included active modes (e.g. walking or cycling as part of a longer public transport journey), were calculated.

### 2.4 Covariates

Participant age (years), highest qualification at baseline (degree or equivalent; less than degree), home ownership at baseline (owns/part-owns; rents/other), car access (access; no access) and commute distance (tertiles) were self-reported. A summary score was calculated from levels of agreement with eight statements about car use based on the Theory of Planned Behaviour, with a higher score indicating participants were more positive towards car use (Supplemental Table S2) (Ogilvie et al., 2016). Subjective assessment of the commuting environment was measured using a summary score calculated from responses to seven statements, e.g. ‘it is pleasant to walk’ and ‘there is little traffic’ (Supplemental Table S2). Using SF-8, a validated measure of health-related quality of life, continuous measures of physical (PCS) and mental (MCS) health were derived and included in the model (Ware et al., 2001). Changes to home and work address were accounted for using a binary variable to indicate whether the postcode sector of both addresses remained unchanged, or whether a change was seen (in either work address, home address or both) between baseline and follow-up.

### 2.5 Analysis

The characteristics of the sample were summarised, and where stratified analyses were indicated, baseline differences between strata were assessed using a Pearson chi-squared test (categorical variables) or one-way ANOVA (continuous variables).

The associations between changes in the workplace environment and changes in the proportion of commutes made by each of the three modes or combinations of mode of transport were examined using a generalised linear model with a logit link and binomial family in order to take account of the bounded nature of the proportion outcomes (i.e. between 0 and 1). The proportion at follow-up was regressed on the proportion at baseline in order to model change in the outcome, which was then presented as a percentage (Twisk, 2013). Change in the physical workplace environment was summarised as a single continuous variable representing the change in the number of physical characteristics. Changes in the social environment were examined for each item separately as this allowed data on the five levels of agreement to be retained and differences between management and colleagues to be explored. Analyses were carried out unadjusted and maximally adjusted, including the variables listed above. Additional analyses were conducted with active travel decomposed into walking and cycling (both exclusively and in combination with other modes) in order to explore differences between these modes. Sensitivity analyses were carried out excluding car parking from the physical characteristics to examine the influence of this factor, which differs from the others as it restricts car use rather than promoting active travel (Knott et al., 2019). In order to examine the impact of participants’ age and gender on any associations, tests for an interaction with these variables were carried out.

## 3 Results

### 3.1 Sample characteristics

Baseline data were provided by 756 individuals who reported being in full-time employment, of whom 227 were lost to follow-up and 110 were excluded due to missing data for one or more variables. A comparison between those included in analyses and those excluded showed that those living in rented accommodation were more likely to be lost to follow-up, but these groups were otherwise broadly similar (Supplemental Table S3). Of 419 commuters who provided complete data at baseline and follow-up, 132 were male and 287 were female (Table 1). The mean age of the sample at baseline was 45 years and was higher in male (48) than female participants (44, P = 0.005). In addition to being disproportionately female, the sample was also of higher socio-economic position than the general population of England, with 67% having a degree and 85% owning their home, neither of which differed by gender (P = 0.300, P = 0.400) (Department for Communities and Local Government, 2017, Office for National Statistics, 2017). The percentage of all commute trips that were made exclusively by private motor vehicle was calculated for each individual; the mean was 29%, compared with 46% of trips made exclusively by active modes and 67% that included the use of active modes. Exclusively active commutes, and those including active travel, formed higher proportions of trips for men than for women (54% versus 43%, P = 0.017 and 74% versus 64%, P = 0.023 respectively). The mean percentage of commutes made exclusively by private motor vehicle was 24% for men and 31% for women (P = 0.069).

**Table 1:** Comparison of the characteristics of female and male commuters.

	Total	Male	Female	p-value*
	N = 419	N = 132	N = 287	
% commutes exclusively by private motor vehicle	29 (40)	24 (36)	31 (42)	0.069
% commutes exclusively active travel	46 (45)	54 (44)	43 (46)	0.017
% commutes including active travel	67 (42)	74 (37)	64 (44)	0.023
Age	45 (11)	48 (11)	44 (11)	0.005
Highest qualification				0.24
Degree	308 (74%)	102 (77%)	206 (72%)	
Less than degree	111 (26%)	30 (23%)	81 (28%)	
Housing tenure				0.32
Rents/other	58 (14%)	15 (11%)	43 (15%)	
Owns/part owns	361 (86%)	117 (89%)	244 (85%)	
Car access				0.71
No car access	48 (11%)	14 (11%)	34 (12%)	
Access to a car	371 (89%)	118 (89%)	253 (88%)	
Tertile of commute distance				0.71
Lowest (mean distance in whole sample 3.4 km)	157 (37%)	52 (39%)	105 (37%)	
Middle (mean distance in whole sample 9.5 km)	130 (31%)	42 (32%)	88 (31%)	
Highest (mean distance in whole sample 29.7 km)	132 (32%)	38 (29%)	94 (33%)	
Change to home and/or work postcode				0.27
Unchanged	316 (75%)	95 (72%)	221 (77%)	
Changed	103 (25%)	37 (28%)	66 (23%)	
Season at follow-up				0.98
Spring	89 (21%)	28 (21%)	61 (21%)	
Summer	115 (27%)	35 (27%)	80 (28%)	
Autumn	122 (29%)	40 (30%)	82 (29%)	
Winter	93 (22%)	29 (22%)	64 (22%)	
Season at baseline				0.13
Spring	131 (31%)	44 (33%)	87 (30%)	
Summer	102 (24%)	33 (25%)	69 (24%)	
Autumn	91 (22%)	34 (26%)	57 (20%)	
Winter	95 (23%)	21 (16%)	74 (26%)	
Mental health summary score (MCS 8)	51 (8)	52 (7)	51 (8)	0.48
Physical health summary score (PCS 8)	54 (6)	55 (5)	54 (6)	0.18

Data are presented as mean (standard deviation) for continuous measures, and n (%) for categorical measures.

Data show baseline values unless otherwise stated.

* P-values for difference between men and women using either a two sample *t*-test (continuous) or a Pearson’s Chi Squared test (categorical).

A change to fewer physical workplace characteristics to promote active travel was reported by 143 participants (34%), with 138 (33%) reporting no change and the same number reporting an increase (Supplemental Table S1). The availability and amount of change differed widely between characteristics. For example, bicycle racks were available for 96% for participants at both time points, with fewer than 4% reporting any change (Supplemental Table S1). In contrast, 24% reported a change in the availability of changing rooms. Baseline data showed that 39% of participants agreed or strongly agreed that many colleagues walked all or part of the way to work, compared with 75% for cycling and 80% for driving (Supplemental Table S1). When asked about senior management, these proportions were 46% for walking or cycling and 69% for driving. For each of the five social environment measures, about half of participants (46%–58%) reported no change, with similar numbers reporting changes to more and less favourable norms in each case. The social environment measure with the greatest level of change was that for colleagues walking to work; 28% reported decreased agreement with this statement and 27% reported increased agreement. The lowest levels of change were seen for colleagues driving, with 21% reporting increased agreement and 21% decreased agreement.

### 3.2 Longitudinal associations with change in mode of travel to work

**Fig. 1:**
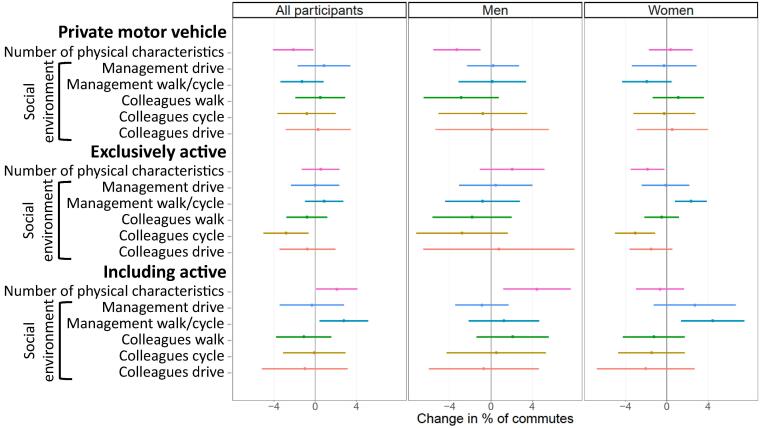
Associations between changes in physical and social characteristics of the workplace and the proportion of commutes by each mode or combinations of mode of transport. Adjusted for: age in years at baseline (continuous), highest qualification at baseline (degree; less than degree), homeownership at baseline (owns/part-owns; rents/other) baseline car access (access; no access), baseline commute distance (tertiles), baseline MCS physical health score (continuous), baseline PCS mental health score (continuous), change in commute i.e. home postcode and/or work postcode changed (stable; changed), season at baseline (spring; summer; autumn; winter), season at follow-up (spring; summer; autumn; winter), a summary score based on the Theory of Planned Behaviour and a summary score of participants’ attitudes towards their environment.

In several analyses of commute mode, an interaction was seen between workplace environment and gender, therefore gender-stratified analyses are presented alongside those for the whole sample; interactions were not seen with age. In adjusted analyses of the physical workplace environment, differences between men and women were apparent. Among men, an increase of one physical characteristic was associated with a 3.3% reduction in the proportion of commutes by private motor vehicle (−3.3%, 95% CI −5.6 to −1.0)) (Table 2 and Fig. 1). In men, a change in the number of physical characteristics was not associated with any changes in the proportion of commutes by exclusively active modes, but an increase of one characteristic was associated with a 4.4% (95% CI 1.2 to 7.7) increase in the proportion of trips which included active modes. No associations with these outcomes were seen in women; indeed, among female participants an increase in physical characteristics was associated with a reduction in trips exclusively by active modes (−1.9%, 95% CI −3.5 to −0.2). Sensitivity analyses which excluded car parking facilities from the exposure measure showed no substantial differences from the main analyses (Supplemental Table S4).

**Table 2:** Associations between changes in physical and social characteristics of the workplace and the proportion of commutes by each mode or combinations of mode of transport.

	All participants				Males				Females			
	Unadjusted		Adjusted		Unadjusted		Adjusted		Unadjusted		Adjusted	
	Change in % (95% CI)	P-value	Change in % (95% CI)	P-value	Change in % (95% CI)	P-value	Change in % (95% CI)	P-value	Change in % (95% CI)	P-value	Change in %	P-value
**Private motor vehicle**												
**Physical characteristics**												
Number of facilities	−2.6% (−4.5% to −0.8%)	**0.005**	−2.1% (−4.1% to −0.2%)	**0.034**	−5.3% (−8.1% to −2.5%)	**<0.001**	−3.3% (−5.6% to −1.0%)	**0.004**	−0.7% (−2.5% to 1.2%)	0.476	0.4% (−1.7% to 2.5%)	0.724
**Social characteristics**												
Management drive	0.6% (−2.1% to 3.4%)	0.655	0.8% (−1.7% to 3.4%)	0.515	2.0% (−2.9% to 6.8%)	0.426	0.2% (−2.3% to 2.7%)	0.877	−0.4% (−3.2% to 2.4%)	0.770	−0.3% (−3.4% to 2.9%)	0.866
Management walk or cycle	−2.0% (−4.0% to 0.0%)	0.052	−1.3% (−3.4% to 0.8%)	0.222	−2.4% (−6.4% to 1.5%)	0.230	0.1% (−3.1% to 3.4%)	0.934	−1.8% (−4.2% to 0.5%)	0.123	−1.9% (−4.3% to 0.4%)	0.110
Colleagues walk	−1.0% (−3.1% to 1.1%)	0.359	0.5% (−1.9% to 2.9%)	0.688	−3.4% (−7.5% to 0.8%)	0.109	−2.9% (−6.5% to 0.7%)	0.119	−0.1% (−2.6% to 2.4%)	0.953	1.1% (−1.4% to 3.6%)	0.378
Colleagues cycle	−1.3% (−4.2% to 1.7%)	0.390	−0.8% (−3.6% to 2.0%)	0.573	−3.0% (−8.2% to 2.2%)	0.260	−0.8% (−5.1% to 3.5%)	0.719	−0.5% (−4.1% to 3.2%)	0.806	−0.3% (−3.2% to 2.7%)	0.870
Colleagues drive	−0.9% (−3.9% to 2.2%)	0.588	0.3% (−2.8% to 3.4%)	0.855	−2.6% (−9.3% to 4.1%)	0.450	0.1% (−5.4% to 5.6%)	0.969	−0.1% (−3.3% to 3.1%)	0.943	0.5% (−2.9% to 4.0%)	0.765
**Exclusively active**												
**Physical characteristics**												
Number of facilities	0.5% (−1.2% to 2.2%)	0.581	0.5% (−1.3% to 2.4%)	0.563	2.7% (−0.2% to 5.6%)	0.064	2.1% (−1.1% to 5.2%)	0.198	−1.2% (−3.1% to 0.7%)	0.205	−1.9% (−3.5% to −0.2%)	**0.025**
**Social characteristics**												
Management drive	−0.5% (−3.0% to 1.9%)	0.664	0.0% (−2.3% to 2.3%)	0.993	0.6% (−4.1% to 5.4%)	0.791	0.5% (−3.1% to 4.0%)	0.799	−1.4% (−3.7% to 0.8%)	0.216	−0.1% (−2.4% to 2.2%)	0.932
Management walk or cycle	0.9% (−1.0% to 2.8%)	0.356	0.9% (−1.0% to 2.7%)	0.348	−0.1% (−4.1% to 3.8%)	0.947	−0.8% (−4.4% to 2.8%)	0.658	1.6% (−0.3% to 3.5%)	0.090	2.3% (0.8% to 3.9%)	**0.003**
Colleagues walk	−0.8% (−2.7% to 1.1%)	0.420	−0.8% (−2.8% to 1.2%)	0.425	−1.2% (−6.0% to 3.6%)	0.627	−1.8% (−5.7% to 2.0%)	0.347	−0.6% (−2.4% to 1.2%)	0.494	−0.5% (−2.2% to 1.2%)	0.560
Colleagues cycle	−2.7% (−4.7% to −0.7%)	**0.007**	−2.8% (−5.0% to −0.6%)	**0.011**	−2.0% (−6.4% to 2.4%)	0.369	−2.8% (−7.2% to 1.6%)	0.217	−3.2% (−5.1% to −1.3%)	**0.001**	−3.1% (−5.0% to −1.1%)	**0.002**
Colleagues drive	−1.0% (−3.9% to 2.0%)	0.523	−0.8% (−3.5% to 1.9%)	0.580	2.0% (−6.2% to 10.2%)	0.627	0.8% (−6.6% to 8.1%)	0.838	−1.6% (−4.0% to 0.8%)	0.183	−1.5% (−3.6% to 0.5%)	0.144
**Including active**												
**Physical characteristics**												
Number of facilities	2.4% (0.6% to 4.1%)	**0.009**	2.1% (0.1% to 4.1%)	**0.037**	5.1% (2.4% to 7.7%)	**<0.001**	4.4% (1.2% to 7.7%)	**0.008**	0.3% (−1.8% to 2.5%)	0.750	−0.7% (−3.0% to 1.7%)	0.576
**Social characteristics**												
Management drive	0.0% (−3.3% to 3.2%)	0.980	−0.3% (−3.4% to 2.8%)	0.842	−2.7% (−7.6% to 2.1%)	0.270	−0.9% (−3.5% to 1.7%)	0.504	1.8% (−2.0% to 5.5%)	0.363	2.7% (−1.3% to 6.7%)	0.181
Management walk or cycle	2.8% (0.6% to 5.1%)	**0.013**	2.8% (0.4% to 5.1%)	**0.021**	2.5% (−1.0% to 6.0%)	0.164	1.3% (−2.2% to 4.7%)	0.472	3.1% (0.2% to 6.1%)	**0.037**	4.5% (1.4% to 7.5%)	**0.005**
Colleagues walk	0.0% (−2.4% to 2.5%)	0.978	−1.1% (−3.8% to 1.6%)	0.420	2.3% (−1.3% to 6.0%)	0.209	2.1% (−1.4% to 5.6%)	0.239	−0.8% (−3.8% to 2.3%)	0.619	−1.3% (−4.2% to 1.7%)	0.411
Colleagues cycle	0.7% (−2.5% to 3.9%)	0.690	−0.1% (−3.1% to 2.9%)	0.961	3.5% (−1.6% to 8.7%)	0.176	0.5% (−4.3% to 5.3%)	0.834	−0.6% (−4.7% to 3.5%)	0.771	−1.5% (−4.7% to 1.8%)	0.370
Colleagues drive	−0.2% (−4.0% to 3.7%)	0.923	−1.0% (−5.1% to 3.2%)	0.640	1.4% (−5.6% to 8.4%)	0.696	−0.7% (−6.0% to 4.6%)	0.798	−0.8% (−5.3% to 3.8%)	0.742	−2.0% (−6.8% to 2.7%)	0.398

Adjusted for: age in years at baseline (continuous), highest qualification at baseline (degree; less than degree), homeownership at baseline (owns/part-owns; rents/other) baseline car access (access; no access), baseline commute distance (tertiles), baseline MCS physical health score (continuous), baseline PCS mental health score (continuous), change in commute i.e. home postcode and/or work postcode changed (stable; changed), season at baseline (spring; summer; autumn; winter), season at follow-up (spring; summer; autumn; winter), a summary score based on the Theory of Planned Behaviour and a summary score of participants’ attitudes towards their environment. CI - Confidence Interval.

In adjusted analyses of changes in the social environment among the full sample, no associations were seen with commutes by private motor vehicle (Table 2 and Fig. 1). A more favourable social environment in respect of colleagues cycling was associated with a reduction in commutes by exclusively active modes in the entire sample (−2.8%, 95% CI −5.0 to −0.6), with the same pattern seen among women (−3.1%, 95% CI −5.0 to −1.1). Although the point estimate was very similar in men, the confidence intervals were wider and encompassed zero (−2.8%, 95% CI −7.2 to 1.6). Among women, a more favourable social environment in respect of management walking or cycling was associated with a 2.3% (95% CI 0.8 to 3.9) increase in commutes by exclusively active modes and a 4.5% (95% CI 1.4 to 7.5) increase in commutes including active modes. These associations were not seen among men.

Analyses with walking and cycling disaggregated were broadly consistent with the main analyses, but with wider confidence intervals (Supplemental Table S5).

## 4 Discussion

### 4.1 Main findings

A physical workplace environment more favourable for active travel was associated with a 4% increase in the proportion of commute trips made by active commuting and a 3% reduction in the proportion of commute trips made by private motor vehicle among men, but this association was not seen in women. The social environment showed a more mixed picture. A more favourable social environment for active commuting among senior management was associated with increased active commuting in women (e.g. a 4% increase in the proportion of commute trips made including active modes). However, a more favourable social environment for cycling among colleagues was associated with reduced active commuting, especially among women (e.g. 3% reduction in the proportion of commutes trips made by exclusively active modes).

### 4.2 Comparison with existing research

Direct comparison with existing research is difficult, due to differences in study design and exposure and outcome measures (Heinen et al., 2013, Petrunoff et al., 2016a, Porter et al., 2019, Scheepers et al., 2014). However, greater numbers of physical workplace characteristics have been cross-sectionally associated with higher levels of cycling (de Geus et al., 2008, Porter et al., 2019), with one study finding no differences between genders, in contrast with our longitudinal findings (Porter et al., 2019). Workplace travel plans have also been found to be associated with increased active commuting (Petrunoff et al., 2016a, Petrunoff et al., 2016b, Petrunoff et al., 2015). Studies investigating specific characteristics have reported mixed findings, with little consensus about what is effective and what role contextual factors might play. Some studies found positive associations between specific characteristics – such as cycle storage, changing facilities and car parking – and commute mode (Heinen et al., 2013, Knott et al., 2019), in some cases using the same dataset used here (Knott et al., 2019). Others have failed to find an association (Bopp et al., 2014, Handy and Xing, 2011, Nehme et al., 2017) – for example with new workplace shower facilities, which one study found were more often used following leisure physical activity than following active commuting (Nehme et al., 2017). Although no association was found in that small study, this is consistent with other evidence suggesting that some workplace characteristics could enable physically active behaviour beyond the commute (Hipp et al., 2017).

Some studies of the workplace social environment have found an association with commuting. For example, a study in the Netherlands found those who felt colleagues expected them to use a car were less likely to be commuter cyclists (Heinen et al., 2013). Another found those that reported that either their “employer encourages active commuting” or a “perception that there are co-workers who actively commute” were more likely to actively commute than those who did not agree with either statement (Kaczynski et al., 2010). However, other studies have failed to find an association between active commuting and either the workplace social environment (such as having co-workers who actively commute) or an overall score for employer support (Bopp et al., 2013).

### 4.3 Interpretation

Increased active commuting was associated with reported increases in the presence of supportive physical workplace characteristics in men, and of a supportive workplace social environment in women. These findings suggest that it may be appropriate to approach the promotion of active commuting differently for men and women, but more research with larger samples and in differing contexts is indicated. One possible explanation for the gender differences we observed was the lower baseline level of active commuting among women, but this was accounted for in analyses. Another potential explanation may lie in unobserved differences between workplaces predominantly employing men or women, which may have led to residual confounding. Our findings echo those of a cross-sectional study of women which found that active commuting was associated with workplace attitudes to active travel but not with bike parking and storage policies (Bopp et al., 2014). A qualitative study comparing attitudes to cycling in three UK workplaces found that changing facilities and cycle security were viewed as more important by men than women (Dickinson et al., 2003). Both studies also found that a strict workplace dress code was a deterrent to cycling among men and women (Bopp et al., 2014, Dickinson et al., 2003). It seems likely that for people who prefer not to get changed at the workplace, being able to work in attire which is convenient for cycling could affect commute choices.

Our finding that a more favourable social environment for cycling among colleagues was associated with reduced active travel is difficult to interpret and we are not aware of other comparable findings. One possible explanation is that witnessing cycling colleagues dressed in sports attire with specialised equipment might reinforce a commonly perceived barrier to cycling, whereby individuals feel they may lack the ability to commute by bicycle (Willis et al., 2015). However, it is uncertain whether a modest change in perceived norms for cycling among colleagues would have a substantial effect in an area with such a high prevalence of cycling as Cambridge.

Existing evidence has suggested that even when a range of actions are taken to encourage active travel, it is difficult to get meaningful behaviour change without taking actions to restrict car use (Petrunoff et al., 2017, Petrunoff et al., 2015). Indeed, it has been shown that less restrictive car parking alone was associated with greater car use (Knott et al., 2019). However, we found that excluding car parking from the workplace exposure measure made little difference to the associations found, suggesting that promoting active travel might be effective even in the absence of restricting car use. Our results therefore support the wider conclusion of existing evidence that the most effective way to increase active travel may be to use a comprehensive package of policies targeting both social and physical environments (Winters et al., 2017). Recent attempts to map a broad range of potential intervention “action types” provide a framework for future researchers and practitioners to identify the potential constituents of any such package (Kelly et al., 2020).

## 5 Strengths and limitations

As workplace characteristics and commuting behaviours were both self-reported and assessed at the same time, albeit at two time points, it remains possible that reverse causality underlies some of our findings. This is plausible, for example in the case of a participant who was unaware of the presence of workplace shower facilities until they engaged in active commuting and needed to use them. A related issue is that participants might not be aware of the presence/absence of a workplace travel plan, even though it may contribute to their ability or willingness to commute actively. Changes in awareness of a characteristic, rather than its existence, might also have contributed to some of the findings seen in this analysis.

The examination of six exposures and three outcomes might have led to type 1 error associated with multiple testing. However, five of the six gender-stratified associations with P < 0.05 had very much smaller P-values (0.001–0.008), which reduces the likelihood of false positive findings. It was not possible to explore the associations of individual workplace characteristics without more participants and/or data points, so their relative importance remains uncertain and a topic for future study. The Commuting and Health in Cambridge study only collected data on the workplace environment in two waves of data; future studies could collect data with greater temporal richness to enable stronger study design and greater statistical power. The comparatively small sample size also prevented more detailed analysis, for example to examine whether car parking moderated the association between the other characteristics and mode change. A study with a larger sample size might also be better placed to explore differences between walking and cycling, which are likely to differ in their determinants (Pikora et al., 2003, Ton et al., 2019). All study participants worked in Cambridge, which may limit the generalizability of both the population and the context. However, by describing the context of our study, we aim to aid interpretation and enable comparison with other studies in different settings to help understand which contextual factors may influence the associations seen. Commuting is only one aspect of everyday mobility and is not equally germane to all in society, so research into travel for other purposes is also indicated.

Additional strengths of this study include the application of a natural-experimental approach using assessment of individual-level changes over time in a longitudinal cohort, after accounting for individual and household characteristics and attitudes to personal travel. In contrast to many studies which have used a single measure of usual commute mode, we were able to use detailed data on participants’ travel behaviour, including journeys combining active travel with other modes. These mixed-mode journeys are potentially important contributors to physical activity and health improvement, and may offer a more realistic alternative to journeys entirely by private motor vehicle for many commuters (Costa et al., 2015).

## 6 Conclusion

This study provides longitudinal evidence for gender differences in the associations between workplace environment and commute mode. A more supportive physical environment was associated with more active commuting in men, while the social environment appeared to have more complex associations that were stronger among women. Although this study was small and geographically circumscribed, its findings propound larger studies in more diverse contexts.

## Declaration of Competing Interest

The authors declare that they have no known competing financial interests or personal relationships that could have appeared to influence the work reported in this paper.
