# Supplementary material for: The social and physical workplace environment and commute mode: A natural experimental study
Source: Prev Med Rep. 2020 Nov 28;20:101260. doi: 10.1016/j.pmedr.2020.101260 (PMC7723790; doi:10.1016/j.pmedr.2020.101260)
Supplement: Supplementary data 1 [file mmc1.docx]

**Supplemental Table S1** – Baseline levels and patterns of changes for each of the workplace characteristics and individual perception measures (n=419)

|  |  | **Baseline N (%)** | | | |  |  | **Change N (%)** |  |
| --- | --- | --- | --- | --- | --- | --- | --- | --- | --- |
| **Physical characteristics*** |  | **Yes** | | **No** | |  | **Decreased**** | **Increased**** | **No change** |
| Summary score |  | Mean = 3.8 (s.d. 1.6) | | | |  | 143 (34%) | 138 (33%) | 138 (33%) |
|  |  |  | | | |  |  |  |  |
| Bicycle rack |  | 409 (98%) | | 10 (2%) | |  | 9 (2%) | 5 (1%) | 405 (97%) |
| Waterproof storage |  | 175 (42%) | | 244 (58%) | |  | 48 (12%) | 54 (13%) | 317 (76%) |
| Changing rooms |  | 223 (53%) | | 196 (47%) | |  | 58 (14%) | 43 (10%) | 318 (76%) |
| Adult cycle training |  | 44 (11%) | | 375 (90%) | |  | 20 (5%) | 12 (3%) | 387 (87%) |
| Bicycle users group |  | 98 (23%) | | 321 (77%) | |  | 24 (6%) | 35 (8%) | 360 (86%) |
| Walk and/or bike to work day |  | 96 (23%) | | 323 (77%) | |  | 35 (8%) | 32 (8%) | 352 (84%) |
| Cycle to work scheme |  | 262 (63%) | | 157 (37%) | |  | 42 (10%) | 43 (10%) | 334 (80%) |
| A travel plan |  | 165 (39%) | | 254 (61%) | |  | 42 (10%) | 41 (10%) | 336 (80%) |
| Car parking |  | 307 (73%) | | 112 (27%) | |  | 26 (6%) | 24 (6%) | 369 (88%) |
| **Social Characteristics** | **Strongly agree** | **Agree** | **Neither agree nor disagree** | | **Disagree** | **Strongly disagree** | **Decreased agreement (less favourable)** | **Increased agreement (more favourable)** | **No change** |
| Many of my colleagues WALK all or part of the way to and from work | 39 (9%) | 127 (30%) | 76 (18%) | | 131 (31%) | 46 (11%) | 116 (28%) | 111 (27%) | 192 (46%) |
| Many of my colleagues CYCLE all or part of the way to and from work | 125 (30%) | 190 (45%) | 46 (11%) | | 43 (10%) | 15 (4%) | 100 (24%) | 95 (23%) | 224 (54%) |
| Many of my colleagues DRIVE to and from work | 151 (36%) | 186 (44%) | 45 (11%) | | 30 (7%) | 7 (2%) | 88 (21%) | 89 (21%) | 242 (58%) |
| Members of senior management WALK OR CYCLE all or part of the way to and from work | 55 (13%) | 138 (33%) | 92 (22%) | | 85 (20%) | 49 (12%) | 102 (24%) | 111 (27%) | 206 (49%) |
| Members of senior management DRIVE to and from work | 110 (26%) | 181 (43%) | 86 (22%) | | 30 (7%) | 12 (3%) | 104 (25%) | 103 (25%) | 212 (52%) |
| * Although some of the characteristics that were considered physical were not strictly physical, the point was to contrast these characteristics with the social context of how others commute.  ** decreased means moving from yes to no; increased means moving from no to yes s.d. = standard deviation | | | | | | | | | |

**Supplemental Table S2 -** Statements derived from the Theory of Planned Behaviour and environmental perception summary scores and their values at baseline

| **Theory of Planned Behaviour** | **Strongly Agree** | **Agree** | **Neither agree  nor disagree** | **Disagree** | **Strongly Disagree** |
| --- | --- | --- | --- | --- | --- |
| *Attitudes* |  |  |  |  |  |
| Overall, it would be good to use a car | 75 (18%) | 52 (12%) | 42 (10%) | 86 (21%) | 164 (39%) |
| It would be pleasant to use a car | 44 (11%) | 60 (14%) | 88 (21%) | 96 (23%) | 131 (31%) |
| *Subjective norms* |  |  |  |  |  |
| Most people who are important to me would support my using a car | 79 (19%) | 83 (20%) | 92 (22%) | 79 (19%) | 86 (21%) |
| Most people who are important to me think I should use a car | 57 (14%) | 44 (11%) | 96 (23%) | 77 (18%) | 145 (35%) |
| *Behavioural control* |  |  |  |  |  |
| It would be easy for me to use a car | 113 (27%) | 107 (26%) | 41 (10%) | 67 (16%) | 91 (22%) |
| I would be able to use a car | 149 (36%) | 143 (34%) | 25 (6%) | 36 (9%) | 66 (16%) |
| *Intent* |  |  |  |  |  |
| I intend to use a car | 88 (21%) | 56 (13%) | 20 (5%) | 56 (13%) | 199 (47%) |
| I am likely to use a car | 99 (24%) | 47 (11%) | 17 (4%) | 63 (15%) | 193 (46%) |
| **Perception of environment** |  |  |  |  |  |
| It is pleasant to walk | 76 (18%) | 144 (34%) | 94 (22%) | 56 (13%) | 49 (12%) |
| The roads are dangerous for cyclists (NW) | 20 (5%) | 91 (22%) | 76 (18%) | 160 (38%) | 72 (17%) |
| There is convenient public transport | 39 (9%) | 116 (28%) | 61 (15%) | 114 (27%) | 89 (21%) |
| There are convenient routes for cycling | 74 (18%) | 180 (43%) | 55 (13%) | 61 (15%) | 49 (12%) |
| There is little traffic | 13 (3%) | 31 (7%) | 40 (10%) | 148 (35%) | 187 (45%) |
| There are no convenient routes for walking (NW) | 85 (20%) | 179 (43%) | 63 (15%) | 41 (10%) | 51 (12%) |
| It is safe to cross the road | 31 (7%) | 195 (47%) | 99 (24%) | 60 (14%) | 34 (8%) |
| NW – Negatively word question, responses reversed for calculation of summary score | | | | | |

**Supplemental Table S3 -** Comparison of the characteristics of those lost to follow-up, those excluded due to missing data, and those included in the analysis

|  | No follow-up | Missing data | Analysis sample | p-value |
| --- | --- | --- | --- | --- |
|  | N=227 | N=110 | N=419 |  |
| % commutes exclusively by private motor vehicles | 32 (42) | 31 (40) | 29 (40) | 0.610 |
| % commutes exclusively by active travel | 45 (46) | 45 (45) | 46 (45) | 0.950 |
| % commutes including active travel | 63 (43) | 64 (41) | 67 (42) | 0.580 |
| Age | 43 (12) | 46 (11) | 45 (11) | 0.005 |
| Highest qualification |  |  |  | 0.36 |
| Degree | 171 (76%) | 73 (68%) | 308 (74%) |  |
| Less than degree | 55 (24%) | 34 (32%) | 111 (26%) |  |
| Housing tenure |  |  |  | 0.007 |
| Rents/other | 53 (24%) | 20 (19%) | 58 (14%) |  |
| Owns/part owns | 172 (76%) | 83 (81%) | 361 (86%) |  |
| Car Access |  |  |  | 0.75 |
| No car access | 29 (13%) | 11 (10%) | 48 (11%) |  |
| Access to a car | 197 (87%) | 98 (90%) | 371 (89%) |  |
| Tertile of commute distance |  |  |  | 0.48 |
| Lowest | 80 (35%) | 39 (36%) | 157 (37%) |  |
| Middle | 65 (29%) | 26 (24%) | 130 (31%) |  |
| Highest | 82 (36%) | 42 (39%) | 132 (32%) |  |
| Change to home and/or work postcode |  |  |  | 0.012 |
| Unchanged |  | 69 (70%) | 316 (75%) |  |
| Changed |  | 30 (30%) | 103 (25%) |  |
| Season at baseline |  |  |  | 0.19 |
| Spring | 57 (25%) | 37 (34%) | 131 (31%) |  |
| Summer | 45 (20%) | 25 (23%) | 102 (24%) |  |
| Autumn | 68 (30%) | 24 (22%) | 91 (22%) |  |
| Winter | 56 (25%) | 24 (22%) | 95 (23%) |  |
| Season at follow-up |  |  |  | 0.69 |
| Spring |  | 23 (21%) | 89 (21%) |  |
| Summer |  | 29 (26%) | 115 (27%) |  |
| Autumn |  | 31 (28%) | 122 (29%) |  |
| Winter |  | 27 (25%) | 93 (22%) |  |
| Mental health score (MCS 8) | 50 (8) | 53 (7) | 51 (8) | 0.017 |
| Physical health score (PCS 8) | 54 (7) | 54 (5) | 54 (6) | 0.71 |

Data are presented as mean (SD) for continuous measures, and n (%) for categorical measures.
Data show baseline values unless otherwise stated

**Supplemental Table S4** – Associations between changes in physical characteristics of the workplace - excluding parking - and the proportion of commutes by each mode or combinations of mode of transport

|  | **All participants** | | | | | **Males** | | | | | **Females** | | | |
| --- | --- | --- | --- | --- | --- | --- | --- | --- | --- | --- | --- | --- | --- | --- |
|  | **Unadjusted** | | | **Adjusted** | | **Unadjusted** | | | **Adjusted** | | **Unadjusted** | | **Adjusted** | |
|  | **Change in %** | | **p-value** | **Change in %** | **p-value** | **Change in %** | **p-value** | | **Change in %** | **p-value** | **Change in %** | **p-value** | **Change in %** | **p-value** |
| **Private motor vehicle** |  | |  |  |  |  |  | |  |  |  |  |  |  |
| **Continuous exposure** |  | |  |  |  |  |  | |  |  |  |  |  |  |
| Physical characteristics | -2.5% (-4.4% to -0.6%) | | **0.009** | -2.0% (-3.9% to 0.0%) | **0.047** | -5.4% (-8.6% to -2.3%) | **0.001** | | -3.1% (-5.5% to -0.6%) | **0.013** | -0.5% (-2.5% to 1.4%) | 0.586 | 0.3% (-1.9% to 2.5%) | 0.779 |
|  |  | |  |  |  |  |  | |  |  |  |  |  |  |
| **Exclusively active** |  | |  |  |  |  |  | |  |  |  |  |  |  |
| **Continuous exposure** |  | |  |  |  |  |  | |  |  |  |  |  |  |
| Physical characteristics | 0.5% (-1.2% to 2.2%) | | 0.574 | 0.6% (-1.1% to 2.4%) | 0.479 | 3.3% (0.6% to 6.1%) | **0.016** | | 3.1% (0.3% to 5.8%) | **0.030** | -1.5% (-3.6% to 0.6%) | 0.154 | -1.7% (-3.4% to 0.0%) | **0.045** |
|  |  | |  |  |  |  |  | |  |  |  |  |  |  |
| **Including active** |  |  |  |  |  |  |  |  |  |  |  |  |  |  |
| **Continuous exposure** |  | |  |  |  |  |  | |  |  |  |  |  |  |
| Physical characteristics | 2.3% (0.4% to 4.2%) | | **0.019** | 2.0% (-0.1% to 4.2%) | 0.058 | 5.2% (2.3% to 8.1%) | **0.000** | | 3.9% (0.8% to 7.1%) | **0.015** | 0.2% (-2.1% to 2.5%) | 0.835 | -0.5% (-2.9% to 2.0%) | 0.707 |
| Adjusted for: age in years at baseline (continuous), highest qualification at baseline (degree; less than degree), homeownership at baseline (owns/part-owns; rents/other) baseline car access (access; no access), baseline commute distance (tertiles), baseline MCS physical health score (continuous), baseline PCS mental health score (continuous),change in commute i.e. home postcode and/or work postcode changed (stable; changed), season at baseline (spring; summer; autumn; winter), season at follow-up (spring; summer; autumn; winter, a summary score based on the Theory of Planned Behaviour &a summary score of participants attitudes towards their environment calculated from levels of agreement with 7 statement with higher score meaning environment perceived as more friendly towards active travel (2 items recoded as they were negatively worded). | | | | | | | | | | | | | | |

**Supplemental Table S5** - Associations between changes in physical and social characteristics of the workplace and the proportion of commutes by each mode or combinations of mode of transport with walking and cycling disaggregated

|  | All participants | | | | Males | | | | Females | | | |
| --- | --- | --- | --- | --- | --- | --- | --- | --- | --- | --- | --- | --- |
|  | Unadjusted | | Adjusted | | Unadjusted | | Adjusted | | Unadjusted | | Adjusted | |
|  | **Change in %** | P-value | **Change in %** | P-value | **Change in %** | P-value | **Change in %** | P-value | **Change in %** | P-value | **Change in %** | P-value |
| **Exclusively walking** |  |  |  |  |  |  |  |  |  |  |  |  |
| **Physical characteristics** |  |  |  |  |  |  |  |  |  |  |  |  |
| Number of characteristics | 0.2% (-0.6% to 1.0%) | 0.554 | 0.1% (-0.6% to 0.9%) | 0.774 | ***** |  | ***** |  | 0.0% (-1.1% to 1.2%) | 0.949 | -0.2% (-1.6% to 1.3%) | 0.816 |
| **Social characteristics** |  |  |  |  |  |  |  |  |  |  |  |  |
| Management drive | -0.3% (-1.6% to 1.0%) | 0.624 | -0.3% (-1.8% to 1.2%) | 0.723 | ***** |  | ***** |  | -0.8% (-2.5% to 0.9%) | 0.344 | -1.4% (-3.3% to 0.6%) | 0.170 |
| Management walk or cycle | 1.0% (0.1% to 2.0%) | **0.038** | 0.5% (-0.5% to 1.5%) | 0.327 | ***** |  | ***** |  | 0.8% (-0.5% to 2.2%) | 0.235 | -0.4% (-1.8% to 1.0%) | 0.549 |
| Colleagues walk | 0.5% (-0.5% to 1.6%) | 0.294 | 0.8% (-0.2% to 1.8%) | 0.129 | ***** |  | ***** |  | 0.8% (-0.5% to 2.1%) | 0.205 | 1.3% (-0.2% to 2.8%) | 0.083 |
| Colleagues cycle | -0.8% (-2.8% to 1.2%) | 0.417 | -1.3% (-3.0% to 0.4%) | 0.130 | ***** |  | ***** |  | -2.2% (-4.0% to -0.3%) | **0.025** | -2.7% (-4.5% to -0.9%) | **0.003** |
| Colleagues drive | -0.6% (-2.1% to 0.9%) | 0.425 | -0.1% (-1.3% to 1.1%) | 0.862 | ***** |  | ***** |  | -0.7% (-2.5% to 1.2%) | 0.483 | -0.3% (-1.9% to 1.3%) | 0.715 |
|  |  |  |  |  |  |  |  |  |  |  |  |  |
| **Including walking** |  |  |  |  |  |  |  |  |  |  |  |  |
| **Physical characteristics** |  |  |  |  |  |  |  |  |  |  |  |  |
| Number of characteristics | 1.2% (-0.4% to 2.8%) | 0.148 | 1.7% (-0.1% to 3.5%) | 0.066 | 1.2% (-0.8% to 3.2%) | 0.231 | 1.7% (-1.9% to 5.4%) | 0.351 | 1.3% (-1.0% to 3.5%) | 0.271 | 2.0% (-0.6% to 4.6%) | 0.128 |
| **Social characteristics** |  |  |  |  |  |  |  |  |  |  |  |  |
| Management drive | 0.7% (-2.1% to 3.5%) | 0.624 | 0.7% (-2.3% to 3.8%) | 0.644 | 0.1% (-2.8% to 2.9%) | 0.973 | -1.8% (-5.0% to 1.3%) | 0.251 | 1.3% (-2.7% to 5.3%) | 0.525 | 1.1% (-3.2% to 5.5%) | 0.613 |
| Management walk or cycle | 2.1% (-0.5% to 4.8%) | 0.116 | 2.3% (-0.1% to 4.6%) | 0.065 | 3.2% (0.8% to 5.5%) | **0.008** | 3.7% (-2.0% to 9.4%) | 0.206 | 1.9% (-1.7% to 5.5%) | 0.310 | 1.9% (-1.4% to 5.2%) | 0.260 |
| Colleagues walk | 0.0% (-2.4% to 2.4%) | 0.988 | -0.7% (-3.3% to 2.0%) | 0.612 | -0.1% (-3.2% to 3.0%) | 0.945 | -3.6% (-7.4% to 0.2%) | 0.066 | 0.2% (-2.8% to 3.2%) | 0.900 | -0.2% (-3.6% to 3.1%) | 0.884 |
| Colleagues cycle | 0.7% (-2.4% to 3.8%) | 0.662 | 0.2% (-2.9% to 3.4%) | 0.878 | 4.6% (0.8% to 8.4%) | **0.019** | 6.1% (1.5% to 10.7%) | **0.009** | -1.0% (-5.1% to 3.1%) | 0.628 | -1.4% (-5.6% to 2.8%) | 0.512 |
| Colleagues drive | -0.5% (-3.8% to 2.8%) | 0.754 | -1.4% (-4.7% to 2.0%) | 0.421 | -1.4% (-4.1% to 1.3%) | 0.306 | -1.1% (-4.7% to 2.6%) | 0.563 | 0.5% (-4.0% to 4.9%) | 0.840 | -1.5% (-5.7% to 2.6%) | 0.472 |
|  |  |  |  |  |  |  |  |  |  |  |  |  |
| **Exclusively cycling** |  |  |  |  |  |  |  |  |  |  |  |  |
| **Physical characteristics** |  |  |  |  |  |  |  |  |  |  |  |  |
| Number of characteristics | 0.5% (-1.3% to 2.4%) | 0.571 | 0.2% (-1.7% to 2.1%) | 0.862 | 2.5% (-0.5% to 5.5%) | 0.096 | 2.0% (-0.9% to 4.9%) | 0.178 | -0.9% (-3.0% to 1.1%) | 0.369 | -2.0% (-4.2% to 0.2%) | 0.075 |
| **Social characteristics** |  |  |  |  |  |  |  |  |  |  |  |  |
| Management drive | -0.2% (-3.1% to 2.6%) | 0.869 | 0.6% (-1.9% to 3.2%) | 0.640 | 0.1% (-4.6% to 4.8%) | 0.977 | 0.8% (-3.2% to 4.7%) | 0.704 | -0.6% (-4.1% to 3.0%) | 0.751 | 0.8% (-2.1% to 3.8%) | 0.578 |
| Management walk or cycle | -0.7% (-3.3% to 2.0%) | 0.630 | 0.0% (-2.4% to 2.4%) | 0.985 | -1.1% (-5.2% to 3.1%) | 0.622 | -1.3% (-5.5% to 2.9%) | 0.558 | -0.5% (-4.0% to 3.1%) | 0.799 | 1.5% (-0.9% to 4.0%) | 0.212 |
| Colleagues walk | -1.3% (-3.4% to 0.9%) | 0.240 | -1.2% (-3.3% to 0.9%) | 0.260 | -0.5% (-5.5% to 4.5%) | 0.837 | -0.2% (-4.6% to 4.1%) | 0.913 | -1.6% (-4.0% to 0.8%) | 0.183 | -1.2% (-3.5% to 1.0%) | 0.284 |
| Colleagues cycle | -2.4% (-5.0% to 0.2%) | 0.065 | -2.4% (-5.4% to 0.5%) | 0.108 | -3.7% (-8.9% to 1.5%) | 0.159 | -4.9% (-9.9% to 0.1%) | 0.054 | -1.7% (-4.4% to 1.1%) | 0.237 | -1.1% (-4.4% to 2.3%) | 0.526 |
| Colleagues drive | -0.5% (-4.0% to 3.1%) | 0.801 | -0.5% (-3.3% to 2.2%) | 0.703 | 2.4% (-5.8% to 10.6%) | 0.560 | 1.5% (-6.4% to 9.4%) | 0.716 | -1.2% (-4.9% to 2.5%) | 0.531 | -1.4% (-3.8% to 1.0%) | 0.268 |
|  |  |  |  |  |  |  |  |  |  |  |  |  |
| **Including cycling** |  |  |  |  |  |  |  |  |  |  |  |  |
| **Physical characteristics** |  |  |  |  |  |  |  |  |  |  |  |  |
| Number of characteristics | 1.3% (-0.3% to 2.9%) | 0.109 | 0.9% (-0.8% to 2.5%) | 0.293 | 3.6% (1.0% to 6.3%) | **0.008** | 1.8% (-0.9% to 4.6%) | 0.190 | -0.4% (-2.2% to 1.5%) | 0.700 | -1.1% (-3.1% to 0.8%) | 0.262 |
| **Social characteristics** |  |  |  |  |  |  |  |  |  |  |  |  |
| Management drive | -0.3% (-3.6% to 2.9%) | 0.836 | -0.1% (-2.7% to 2.6%) | 0.966 | -2.3% (-7.6% to 2.9%) | 0.381 | -1.0% (-4.0% to 2.1%) | 0.536 | 1.0% (-2.6% to 4.5%) | 0.594 | 1.6% (-1.9% to 5.2%) | 0.375 |
| Management walk or cycle | 0.3% (-2.4% to 3.1%) | 0.819 | 0.6% (-2.2% to 3.4%) | 0.690 | 0.5% (-3.4% to 4.4%) | 0.803 | 0.4% (-4.5% to 5.3%) | 0.868 | 0.2% (-3.5% to 3.9%) | 0.917 | 1.6% (-1.8% to 5.1%) | 0.353 |
| Colleagues walk | 0.1% (-2.2% to 2.5%) | 0.927 | 0.4% (-2.1% to 2.9%) | 0.749 | 2.4% (-1.2% to 6.0%) | 0.191 | 2.6% (-1.1% to 6.3%) | 0.168 | -0.7% (-3.6% to 2.2%) | 0.625 | 0.0% (-2.8% to 2.9%) | 0.976 |
| Colleagues cycle | -0.6% (-3.4% to 2.2%) | 0.666 | -1.1% (-4.0% to 1.7%) | 0.431 | -0.7% (-6.7% to 5.2%) | 0.810 | -4.2% (-9.6% to 1.3%) | 0.133 | -0.6% (-3.5% to 2.2%) | 0.659 | -0.6% (-3.6% to 2.4%) | 0.684 |
| Colleagues drive | 1.4% (-2.0% to 4.8%) | 0.426 | 1.3% (-1.9% to 4.6%) | 0.425 | 3.0% (-3.6% to 9.5%) | 0.379 | 1.0% (-4.6% to 6.6%) | 0.729 | 1.0% (-2.8% to 4.9%) | 0.600 | 0.4% (-3.6% to 4.4%) | 0.856 |
| Adjusted for: age in years at baseline (continuous), highest qualification at baseline (degree; less than degree), homeownership at baseline (owns/part-owns; rents/other) baseline car access (access; no access), baseline commute distance (tertiles), baseline MCS physical health score (continuous), baseline PCS mental health score (continuous),change in commute i.e. home postcode and/or work postcode changed (stable; changed), season at baseline (spring; summer; autumn; winter), season at follow-up (spring; summer; autumn; winter), a summary score based on the Theory of Planned Behaviour and a summary score of participants’ attitudes towards their environment.  ***** unstable estimates for commutes made exclusively by walking are not presented | | | | | | | | | | | | |

**Table S6 - Comparison of the walking and cycling commuting of female and male commuters**

|  | **Total** | **Male** | **Female** | **p-value** |
| --- | --- | --- | --- | --- |
|  | **N=419** | **N=132** | **N=287** |  |
| % of commutes by walking only | 6 (21) | 6 (22) | 6 (20) | 0.800 |
| % of commutes including walking | 21 (37) | 19 (37) | 22 (37) | 0.440 |
| % of commutes by cycling only | 40 (44) | 48 (44) | 37 (44) | 0.017 |
| % of commutes including cycling | 46 (45) | 55 (44) | 42 (45) | 0.006 |

Data are presented as mean (SD) for continuous measures.
Data show baseline values
